# Transcutaneous Spinal Cord Stimulation Attenuates Blood Pressure Drops in Orthostasis

**DOI:** 10.3390/life13010026

**Published:** 2022-12-22

**Authors:** Natalia N. Beliaeva, Tatiana R. Moshonkina, Oleg V. Mamontov, Elena N. Zharova, Heber Ivan Condori Leandro, Nigar Z. Gasimova, Evgeny N. Mikhaylov

**Affiliations:** 1 Almazov National Medical Research Centre, 2 Akkuratova Str., St. Petersburg 197341, Russia; 2 Pavlov Institute of Physiology, Russian Academy of Sciences, 6 Makarova enb., St. Petersburg 199034, Russia

**Keywords:** spinal cord stimulation, neuromodulation, orthostatic hypotension

## Abstract

Orthostatic hypotension is a complex medical problem with various underlying pathogenic mechanisms and limited modalities for its correction. Since transcutaneous spinal cord stimulation (t-SCS) leads to immediate blood pressure (BP) elevation in a supine position, we suggested that t-SCS may attenuate blood pressure drops in orthostasis. We aimed to evaluate the hemodynamic effects of t-SCS during tilt testing in a feasibility study in three patients with documented orthostatic hypotension. Four sessions on two different days of tilt testing on and off t-SCS were performed on each patient. While tilting with t-SCS off showed typical significant BP drops in every patient, active t-SCS resulted in systemic vascular resistance (SVR) elevation in all patients and significantly higher values of systolic and diastolic BP in two patients. T-SCS requires further investigation on a larger patient population. However, our preliminary results demonstrate its ability for SVR and BP elevation in subjects with severe orthostatic hypotension.

## 1. Introduction

Hypotension is highly prevalent and might be associated with endocrine, central, and peripheral neurological disorders. One of the major issues in cardiology practice is orthostatic hypotension (OH), when standing is followed by a significant blood pressure drop (>20 mm Hg for systolic blood pressure) leading to dizziness, fatigue, or even syncope. Causes for OH are multifactorial and include central and peripheral neuropathies. Additionally, OH may be caused by dehydration, pharmacological treatment for cardiovascular diseases (using beta-blockers or other antiarrhythmic medications), and intoxication, among other factors.

A number of treatments have been suggested for OH, such as midodrine, fludrocortisone, and pyridostigmine, but they fail in a significant number of cases. Fluid and salt consumption, physical counterpressure maneuvers, and exercise training are useful for OH in most patients. However, despite the above-mentioned approaches, OH remains a challenging condition for some very symptomatic patients, and it severely impacts their quality of life.

Recently, a few small studies reported on the possibility of spinal cord stimulation to increase blood pressure in subjects with spinal cord injury or multiple-system atrophy and OH [1,2]. Our group demonstrated that transcutaneous spinal cord stimulation (t-SCS) increased blood pressure and enhanced atrioventricular conduction in subjects without neurological impairment and/or structural heart disease [3]. With this approach, t-SCS using a very high-frequency sub-threshold current is being used for transient blood pressure elevation mediated through autonomic nervous system modulation.

Despite the promising messages from the reports of epidural and t-SCS in patients with OH against the background of severe neurology deficit, data on the potential effects of t-SCS on blood pressure correction in otherwise healthy subjects are lacking. OH can be reproducibly induced by verticalization in standard settings (passive orthostatic test = tilt test) in order to assess the effect of an intervention on blood pressure correction.

In this feasibility study, we seek to explore the potential effects of t-SCS on blood pressure in patients with documented orthostatic hypotension during tilt testing.

## 2. Materials and Methods

We enrolled three patients with symptomatic orthostatic hypotension and reproducibly documented significant blood pressure drops during tilt-testing (at least two tests performed on different days with systolic a blood pressure decrease ≥20 mm Hg within the first 10 min after tilting). After inclusion, each patient underwent tilt testing with t-SCS and without stimulation.

### 2.1. Ethical Concerns

The pilot study was approved by an Internal Review Board and the local ethics committee of the Almazov National Medical Research Centre; the patients provided written consent for the procedure.

### 2.2. Study Population

Patient #1 was a 27-year-old woman with a history of recurrent syncope due to mild autonomic dysfunction and orthostatic hypotension paired with an excessive increase in heart rate in orthostasis. OH in this patient was primarily caused by an excessive decrease in stroke volume due to blood depositing and a decrease in preload, leading to an excessive increase in HR while standing. A connective tissue disorder was suspected as the underlying pathology, but not all criteria had been met for a final diagnosis yet. No medications were taken. The patient had no concomitant diseases.

Patient #2 was a 60-year-old man with amyloid polyneuropathy leading to severe orthostatic hypotension and chronotropic dysfunction due to the lesion in the afferent and efferent links of the arterial baroreflex, while the central link remained intact. The patient had no signs of amyloid cardiomyopathy. Ongoing pharmacological therapy: fludrocortisone 0.1 mg per day. The patient had no concomitant diseases.

Patient #3 was a 56-year-old woman with a history of severe orthostatic hypotension due to vasomotor and chronotropic dysfunction who had been treated for primary autonomic dysfunction (severe orthostatic hypotension and chronotropic incompetence accompanied by incomplete bladder emptying, impaired sweating, and sleep disorders). No signs of neuropathy were found, suggesting a central cause for OH. Ongoing pharmacological therapy: fludrocortisone 0.1 mg per day. The patient had no concomitant diseases.

### 2.3. Electrical Transcutaneous Spinal Cord Stimulation during Tilt-Testing

The procedures were performed on two different days: the “index day” and after a week at day “index+7”, two tests per day: with and without t-SCS, alternating consequence. The tests were carried out at the same time of day. The day of the test, the patients had their usual activities, had a light meal, and took all of the usual medication.

The t-SCS methodology was described earlier [3,4]. Briefly, the preparation for the procedure included electrocardiography (ECG) electrode placement and adhesive stimulation patches placement (32 mm foam round electrodes, (FIAB, Italy). The stimulation electrodes were attached to the back skin at the levels of T1, T5, and T7 segments; the reference patches (anodes, 80 mm foam electrodes, FIAB, Italy) were placed on the iliac crests bilaterally). Then, electromyography electrodes were also attached to the patient’s skin bilaterally over the following muscles, innervated by the studied spinal cord segments: mm. trapezius, rhomboideus, and latissimi dorsi. To evaluate the muscle stimulation threshold, as muscle contraction due to a simple spinal motor reflex activation is one of the most noticeable effects of SCS, repetitive single stimuli delivery was performed with a gradual increase in the electrical current (Neurosoft stimulator, Ivanovo, Russia). Once muscle contraction was documented by electromyography (Neuro-MEP registrator, Ivanovo, Russia), the threshold was defined. The electrical current for further high-frequency stimulation (t-SCS with 30 Hz bipolar-modulated (5 kHz) current; NeoStim-3, Cosyma, Moscow, Russia) was set at 80% of the threshold value in order to avoid muscle contractions and prevent possible inconvenient sensations.

T-SCS was delivered to all three levels of stimulation simultaneously. According to previous findings, high-frequency spinal cord stimulation with modulated current might cause sympathetic preganglionic neural excitation [5].

### 2.4. Tilt Testing

Next, 75-degree table verticalization was performed, and the test was held for up to 10 min or until the symptoms of orthostatic incompetence or a severe decrease in systolic blood pressure appeared (>20 mm Hg). Beat-to-beat blood pressure recording was carried out using the Finometer-Pro BP monitor (Finapres Medical Systems, The Netherlands) with parallel electrocardiogram (ECG) recording. Stroke volume (SV), systemic vascular resistance (SVR), total arterial compliance, and cardiac output (CO) were calculated using integrated software.

### 2.5. Sham Procedure

When a sham procedure was scheduled during tilting, all of the preparations were the same, including the muscle stimulation threshold evaluation; an operating physician loudly announced “stimulation is on”, but high-frequency stimulation was not delivered, and 30 s later, the table was tilted by 75 degrees. The test and measurements were performed as during t-SCS.

Both t-SCS and sham stimulation during tilt-testing were repeated one week after the first procedure in reverse order. The patients were not aware of the order and type of the procedure (t-SCS or sham).

## 3. Results

**Figure 1:**
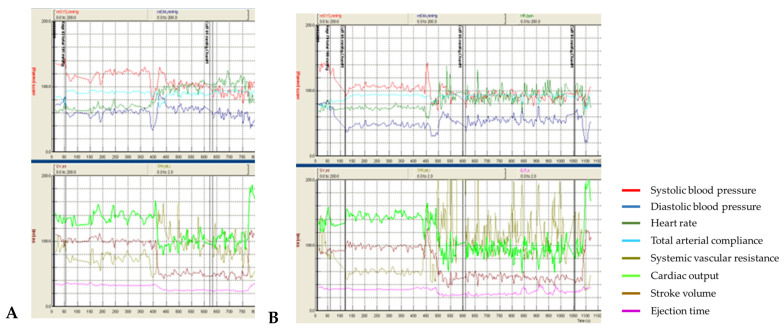
Panel (**A**) shows the dynamics of systolic blood pressure, diastolic blood pressure, heart rate total arterial compliance, systemic vascular resistance, cardiac output, stroke volume, and ejection time during the sham procedure. The transition to orthostasis time takes approximately 400–410 ss. Panel (**B**) shows the dynamics of the same parameters during the test with t-SCS. The transition to orthostasis time lasts approximately 440–470 ss. The flatter slope of the blood pressure curves and a greater level of systemic vascular resistance during t-SCS are noticeable.

Patient #1 had no orthostatic complaints during all 4 tests. However, an excessive decrease in blood pressure in orthostasis, corresponding to relatively mild orthostatic hypotension, was detected. In tests with t-SCS (Figure 1B), a reproducible effect of t-SCS was observed, which consisted of reducing the orthostatic decrease in blood pressure and increasing the systemic vascular resistance, compared to the sham procedures (Figure 1A).

A median difference in systolic BP drops between tests with and without stimulation was 13 mmHg (−22.3 ± 1.8 mmHg with t-SCS vs. −34.4 ± 0.5 mmHg without t-SCS), diastolic BP—12.9 mmHg (+3.8 ± 7.1 mmHg vs. −5.5 ± 1.9 mmHg). The increase in SVR was significantly higher in the t-SCS tests (+1.65 ± 0.3 medical units (MU = mmHg/mL/s) vs. +0.56 ± 0.19 MU).

In the tests with stimulation, there was a more pronounced drop in CO (−71.5 ± 1.4% from the baseline vs. −44.2 ± 15.2%) in orthostasis, while there was no effect on the stroke volume drop in orthostasis: the stroke volume drop at the “index” day was −68.6 mL in the sham procedure and −75.3 mL during stimulation. On the “index+7” day, the stroke volume decreased by 56.5 mL in the sham procedure and by 55,0 mL during stimulation.

The increase in heart rate in orthostasis was slightly more prominent on stimulation (+43.2 ± 4.3 bpm vs +40.8 ± 4.7 bpm).

Thus, in this young patient with mild autonomic dysfunction and orthostatic hypotension, an increase in SVR was detected during stimulation, resulting in a decrease in orthostatic blood pressure drop. However, there was no significant increase in heart rate (there was a timely relationship between an increase in heart rate and the CO drop but not at the beginning of stimulation) and stroke volume. CO, directly dependent on heart rate and stroke volume and inversely dependent on SVR, decreased more significantly in both tests with stimulation.

**Figure 2:**
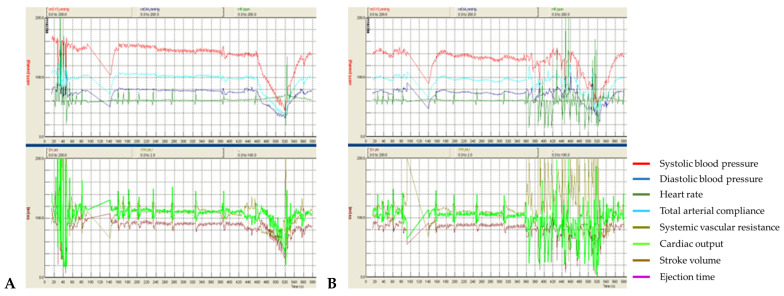
Panel (**A**) shows hemodynamic changes during the test without stimulation, while panel (**B**) shows the test carried out during stimulation. Transition to orthostasis starts in both panels at 460 ss. The increase in SVR is noticeable, with its peak in absolute values amounting to 3.29 MU on stimulation vs. 0.969 MU without stimulation in the presented test.

In patient #2, both sham tests without t-SCS on the “index” and “index+7” days resulted in a severe drop in systolic and diastolic BP following tilting within the first minute (Figure 2A). However, t-SCS attenuated BP drops in both tests (Figure 2B).

A median difference in systolic BP drops between tests with and without stimulation was 15 mmHg (−75.0 ± 10.7 mmHg vs. −90.9 ± 0.3 mmHg, respectively), diastolic—12.2 mmHg (−32.0 ± 0.6 mmHg vs. −44.2 ± 2.1 mmHg, respectively). T-SCS caused an increase of SVR in orthostasis (+3.23 ± 1.67 MU), while in the sham procedures SVR in orthostasis decreased (−0.20 ± 0.08 MU).

In the test with stimulation, a slightly more pronounced increase in heart rate in orthostasis was observed (+6.8 ± 7.8 bpm vs. +3.7 ± 0.0 bpm), while stroke volume (−63.4 ± 18.8 mL vs. −38.5 ± 5.2 mL) and cardiac output (−80.1 ± 11.0% from the baseline vs. −38.0 ± 7.4%) decreased.

In this patient with severe orthostatic hypotension caused by amyloid neuropathy, with the sharp decrease in vasomotor regulation, SVR in orthostasis, and prominent chronotropic dysfunction, an increase in SVR was detected during stimulation, and the BP drop in orthostasis was attenuated. However, even with stimulation, orthostatic hypotension remained severe due to a lack of positive inotropic and chronotropic changes, which presumably resulted in increased afterload and a more prominent CO drop.

**Figure 3:**
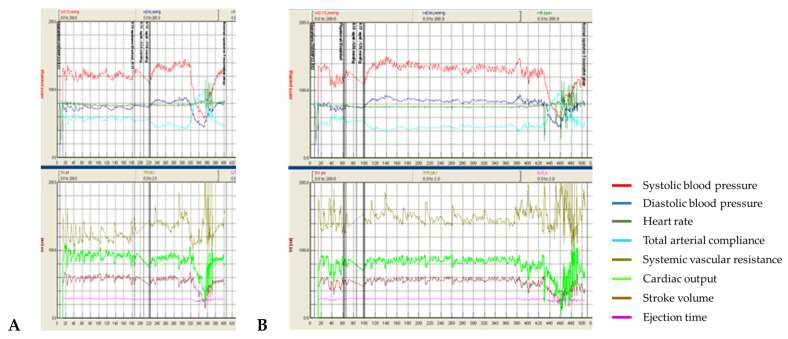
Panel (**A**) shows the dynamics of the parameters measured in the test without stimulation. The transition to orthostasis time is approximately 300–310 ss. Panel (**B**) demonstrates the results obtained during the test with stimulation. The transition to orthostasis time is approximately 420–430 ss. The dynamics of BP and heart rate remain the same. However, the increase in SVR is clearly seen, with its peak in absolute values amounting to 2.23 MU on stimulation vs 1.54 MU without stimulation in the present test.

Patient #3 in sham procedures developed severe orthostatic hypotension within the first minute (Figure 3A). However, despite the increase in SVR during stimulation (Figure 3B), which amounted to median values of +0.73 ± 38 MU vs. +0.26 ± 0.21 MU, there was no increase in BP during t-SCS and an even more pronounced decrease (−78.1 ± 9.2 mmHg vs. −70.0 ± 8.5 mmHg for systolic BP, −39.1 ± 2.9 mmHg vs. −32.3 ± 5.2 mmHg) for diastolic BP. In this patient, there was no significant change in the CO and SV drop during stimulation, and a mildly more prominent increase in HR was observed (+7.2 ± 0.6 bpm vs. +5.2 ± 0.7 bpm).

In this patient with a diagnosis of idiopathic autonomic dysfunction, despite the increase in SVR during stimulation, there was no increase in blood pressure in orthostasis.

## 4. Discussion

The hypotensive orthostatic reaction may occur due to many mechanisms, including drug-induced hypotension, when OH is associated with the reduction in circulating blood volume, or OH associated with primary (idiopathic autonomic dysfunction, multiple-system atrophy, Parkinson’s disease, or dementia with Lewy bodies) or secondary (diabetes mellitus, amyloidosis, spinal cord injury, autoimmune autonomic neuropathy, paraneoplastic autonomic neuropathy, or renal failure) autonomic dysfunction (neurogenic OH).

Neurogenic OH is usually the result of the inadequate release of norepinephrine due to autonomic dysfunction. Neurogenic OH may not be present solely as a disease of BP; it can also be associated with hypertension (for example, in a supine position). Such dysfunction occurs either due to the impairment of the central nervous pathways that regulate the sympathetic nervous system (primary neurogenic OH) or due to the insufficient activation of vascular adrenoreceptors due to degenerative changes in postganglionic sympathetic neurons (secondary neurogenic OH).

Thus, the prevalence of OH in various groups of patients and the multilevel nature of this problem make it extremely relevant. However, OH remains challenging for clinical management. The lack of options for pharmacological or interventional treatment with sufficient evidence in this group of patients leads to a further search for therapeutic approaches.

Patients with spinal cord injury in the cervical or upper thoracic segments constitute a separate group of patients with secondary autonomic insufficiency. In complete spinal cord injury, the loss of the efferent pathway from the brain stem nuclei neurons to the preganglionic neurons of the spinal cord leads to the impairment of blood pressure regulation. The use of electrical spinal cord stimulation in this group of patients for motor rehabilitation purposes was associated with a rise in BP [6] and its stabilization in orthostasis [1].

There are two types of spinal cord electrical stimulation: invasive epidural, when stimulation is delivered via electrodes in the epidural space, and non-invasive transcutaneous stimulation (t-SCS), as described in this and previous reports, when patch electrodes are placed on the skin paravertebrally. Epidural stimulation as an invasive type of intervention can be associated with complications, such as infection of the stimulator pocket, hematoma, traumatization, and electrode migration. T-SCS is free of those limitations, and it has been shown that motor responses in both types of stimulation are identical, have the same latency, and have similar patterns that indicate the activation of the same neural structures [4]. Therefore, the transition from epidural to non-invasive spinal cord stimulation is considered acquitted both in terms of safety and effectiveness.

As a hypothesis explaining the compensating effect of t-SCS on an excessive blood pressure decrease in orthostasis after spinal cord injury, Philips et al. suggested the excitation of propriospinal and sympathetic preganglionic neurons, either directly through the stimulation reaching the spinal cord or by the preferential excitation of large-diameter sensory axons. The mechanism is very similar to that reported in epidural SCS studies [1]. Interestingly, in the study carried out by Philips et al. [1], no effect of t-SCS on SV and HR was found, and neither was it in our patients.

Squair et al. determined, based on the preclinical experimental data, the so-called hemodynamic hotspots—segments T11-T13 with the major density of sympathetic preganglionic neurons, located in posterior roots, and responsible for the pressor effect of invasive spinal cord stimulation, which were proved with the ablation of those neurons, leading to the suppression of pressor responses. The further mechanism of the pressor effect of spinal cord stimulation includes the involvement of neurons located in splanchnic sympathetic ganglia. These efferent neurons are being activated by epidural SCS, with the release of norepinephrine inducing vasoconstriction through the activation of α1 receptors [5].

Similar effects of invasive spinal cord stimulation were observed in the clinical case of implantation of an epidural stimulator at the T-11 segment level in a patient with multiple-system atrophy [2].

Recently, we conducted a pilot study of t-SCS in subjects without structural heart disease undergoing electrophysiological testing, with an assessment of hemodynamic parameters. T-SCS was performed in nine patients at levels T1, T7, and T11. During T1 and T7 stimulation, there was a short-term increase in BP and a reduction in the refractory period of the atrioventricular node [3].

The results obtained in the cases described in this article, namely, an increase in SVR, leading in some cases to attenuation of BP drop in orthostasis, are consistent with previous experimental results by other authors and the hypotheses described above, explaining the vasopressor effects of t-SCS. However, an important difference is the less prominent effect of t-SCS on BP, which may be conditioned by the use of an electrical current with amperage below the motor threshold, whereas in the discussed studies on patients with spinal cord injury and multiple-system atrophy, electrical current at the motor threshold level was used. Among the other reasons for the detected difference in effects, we suggest the contribution of the “muscle pump” effect—an increase in venous return during stimulation with the amperage of the motor threshold.

For the manifestation of the effect of spinal cord stimulation, since stimulation most likely involves spinal cord neurons, the genesis of orthostatic hypotension may be important, including a lesion of the efferent baroreflex pathways in patients with neuropathy, whereas patients with spinal cord injury have a lesion at the central level and preserved peripheral nerves, which allow for the excitation of sympathetic preganglionic neurons, which can then conduct to the periphery.

The absence of an increase in sympathetic influence on the heart and its inotropic and chronotropic functions (that is, the excitation of β1 adrenoreceptors) in the presence of signs of an increase in sympathetic vascular tone (excitation of α1 adrenoreceptors) remains unclear. The increase in heart rate is most likely compensatory and is not directly related to stimulation, based on timing.

Partial sympathetic nervous system activation, leading to an increase in afterload (increased SVR and BP) in the absence of an increase in inotropic function (SV) and heart rate, probably causes a decrease in cardiac output during stimulation.

## 5. Study Limitations

Our preliminary report has some certain limitations. First of all, t-SCS has been used in three patients only, and the small number of patients does not allow one to make firm conclusions about the potential reproducibility of our results. However, we consider the case series illustrative and the first results very promising since repeated tilt tests provide sustainable results. The other limitation is the heterogeneity of the studied patients, and the hemodynamic effects of SCS may vary in patients with different orthostatic insufficiency etiologies. Presumably, t-SCS is likely to be more effective in patients with central genesis of orthostatic hypotension since the structures responsible for the pressor effect of SCS are intact.

## 6. Conclusions

Non-invasive spinal cord stimulation in patients with OH requires further research in randomized studies. However, our preliminary findings suggesting the positive effects of t-SCS on SVR and BP seem promising.

## Data Availability

Data related to this study can be provided by the corresponding authors on request.
